# Compensated Row-Column Ultrasound Imaging System Using Fisher Tippett Multilayered Conditional Random Field Model

**DOI:** 10.1371/journal.pone.0142817

**Published:** 2015-12-11

**Authors:** Ibrahim Ben Daya, Albert I. H. Chen, Mohammad Javad Shafiee, Alexander Wong, John T. W. Yeow

**Affiliations:** Department of Systems Design Engineering, University of Waterloo, Ontario, Canada; University of Chicago, UNITED STATES

## Abstract

3-D ultrasound imaging offers unique opportunities in the field of non destructive testing that cannot be easily found in A-mode and B-mode images. To acquire a 3-D ultrasound image without a mechanically moving transducer, a 2-D array can be used. The row column technique is preferred over a fully addressed 2-D array as it requires a significantly lower number of interconnections. Recent advances in 3-D row-column ultrasound imaging systems were largely focused on sensor design. However, these imaging systems face three intrinsic challenges that cannot be addressed by improving sensor design alone: speckle noise, sparsity of data in the imaged volume, and the spatially dependent point spread function of the imaging system. In this paper, we propose a compensated row-column ultrasound image reconstruction system using Fisher-Tippett multilayered conditional random field model. Tests carried out on both simulated and real row-column ultrasound images show the effectiveness of our proposed system as opposed to other published systems. Visual assessment of the results show our proposed system’s potential at preserving detail and reducing speckle. Quantitative analysis shows that our proposed system outperforms previously published systems when evaluated with metrics such as Peak Signal to Noise Ratio, Coefficient of Correlation, and Effective Number of Looks. These results show the potential of our proposed system as an effective tool for enhancing 3-D row-column imaging.

## Introduction

Ultrasound imaging is a valuable tool in non destructive testing [1, 2], with applications ranging from detection of material defects to object and foreign body detection. 3-D ultrasound imaging offers the possibility of accurately generating certain material properties that could be useful to material scientists [3]. 3-D ultrasound imaging could also be useful in medical imaging: it is difficult to image the same slice in 2-D for the purpose of follow up studies, and viewing of anatomy using a 2-d imaging device requires a great deal of skill and experience [4].

When designing 3-D ultrasound imaging systems, electronic beam-steering with a fixed transducer is preferred over a mechanically moving one; as mechanical motion introduces unwanted artifacts and increases image acquisition time. A mechanically fixed 2-D array of transducers is capable of acquiring high quality 3-D ultrasound image [5, 6]. However, in a fully addressed 2-D array, the total number of elements scales with the square of the number of elements in each dimension [7]. This leads to an impractical number of interconnections (since every individual element needs to be addressed) and a significant amount of data to handle, posing a challenge both in terms of real-time data processing and the actual fabrication of connections [8–10].

**Fig 1:**
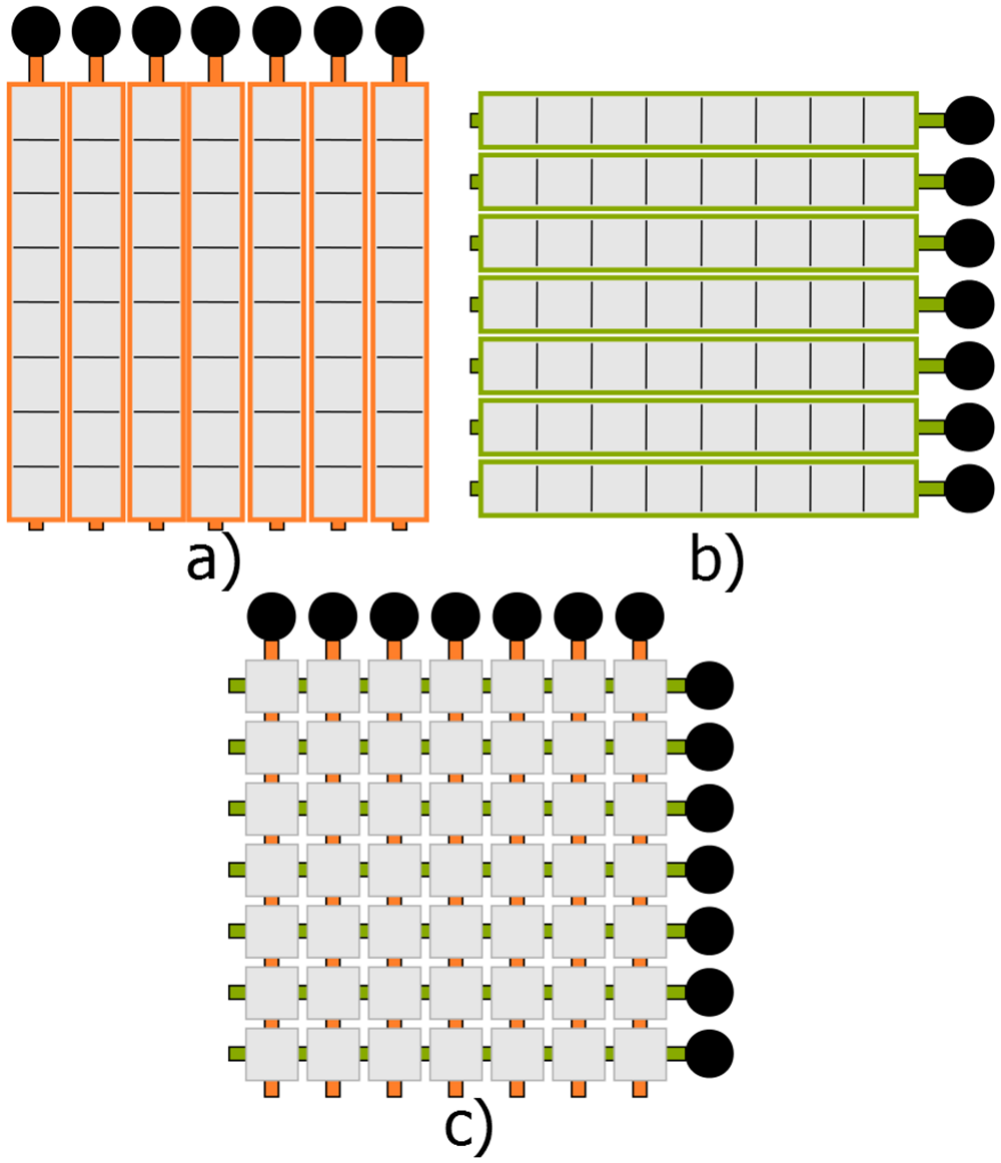
Visualization of a row-column array. (a) shows *N* column arrays with *N* connections, (b) shows *N* row arrays with *N* connections, (c) shows the row column arrangement with 2*N* connections.

A technique proposed by [11], which suggests the use of a pair of orthogonally positioned 1-D arrays of rows and columns (Fig 1) where one is responsible for transmit beamforming and the other for receive beamforming, directly addresses these issues. A line of focus, adjustable in both depth and azimuth, is generated in a manner similar to 1-D transmit beamforming by the column array. Receive beamforming is achieved when the sound reflected from the object being imaged is received by the row array. Receive 1-D array performs software beamforming so a b-mode image can be reconstructed in each transmit event, forming a complete 3-D images after the final transmit event.

An *N* × *N* 2-D array can be designed with only 2*N* connections when this row-column technique is used, as opposed to *N* ^2^ connections with the fully addressed one. Furthermore, according to [7], for any fixed number of active elements, the row-column addressing scheme produces higher quality ultrasound images as compared to the fully addressed one.

**Fig 2:**
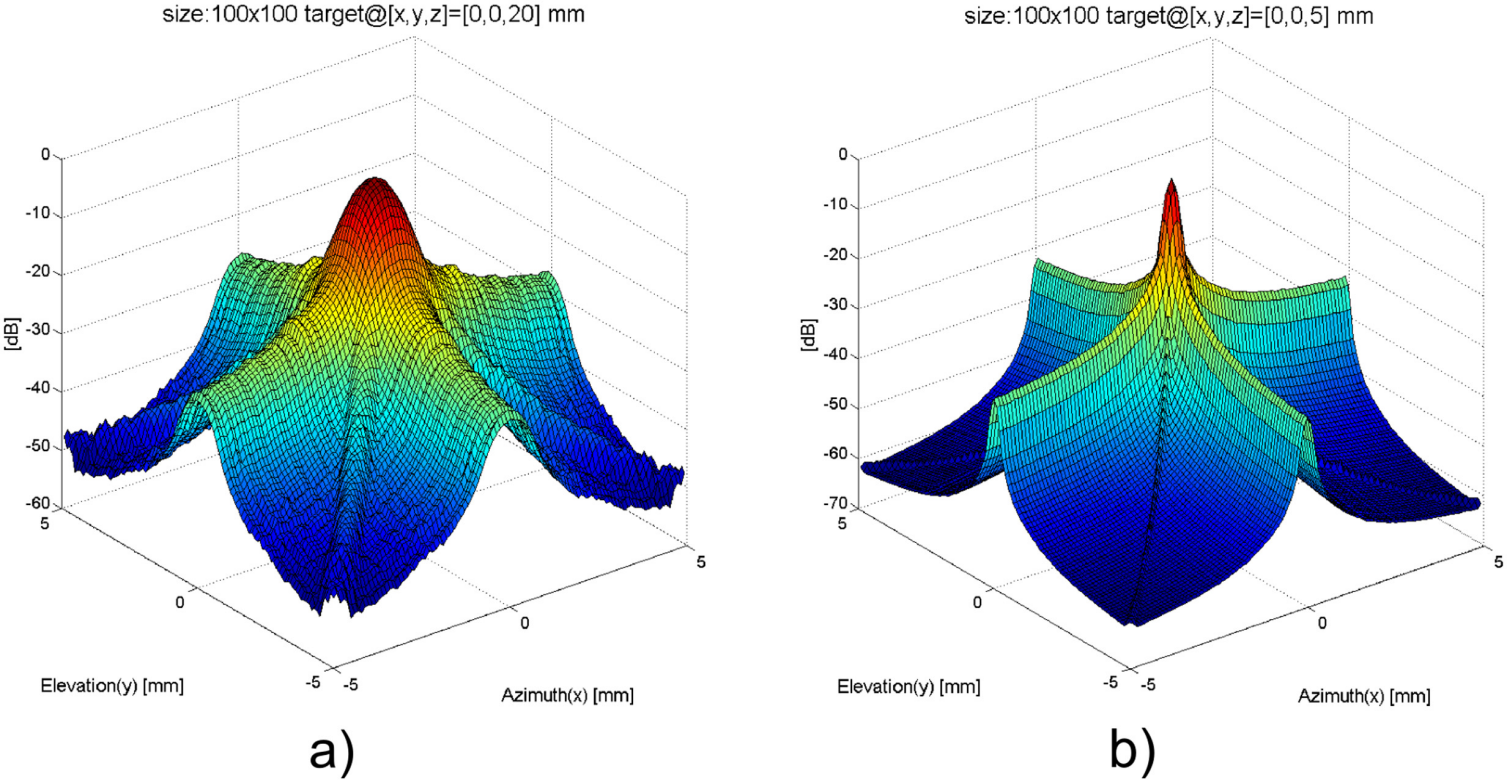
Two-way radiation pattern (i.e. PSF) of a 5 mm × 5 mm, 32 × 32 elements row-column array. The -6 dB resolution weakens from 0.5 mm in spot size to 0.9 mm as the focusing and scatterer moves from 5 mm to 20 mm away from the aperture. Side lobes can be seen below -30 dB. Side lobe shape is highly influenced by the natural focusing tendency of the row-column beamforming method.

Since row-column only focuses in azimuth for transmit and elevation for receive, beamforming relies on natural focusing for elevation during transmit and azimuth during receive. Therefore, the focusing power for row-column beamforming scheme is limited. Pressure near the transducer significantly varies as sound emitted from different parts of the transducer interferes constructively and destructively. The variation in pressure decreases as sound travels away from the transducer, creating a varying beam profile that changes the response of the imaging system with depth. Fig 2 shows the elevation beam profiles at two different depths away from a target point. The varying beam profile poses a challenge when it comes to image reconstruction. The row-column method also suffers from ghost effects in the point spread function (PSF) caused by edge waves [12]. Taking into account a spatially dependant point spread function (and its ghost artifacts) into the reconstruction framework is highly desired to improve image quality.

3-D reconstruction of ultrasound images poses some other interesting challenges. Real-time 3-D imaging requires finite transmit events, meaning that the readings of the scanner are inherently sparse; what needs to be done is recover the full 3-D image of the target object from incomplete data. Another challenge is the nature of speckle noise that is inherent to ultrasound images, and how it should be modeled. While the issues of sparsity and noise have been addressed in literature, the problem with spatially dependant PSF for row-column ultrasound imaging has not been fully explored. In the majority of literature related to row-column ultrasound imaging systems, image reconstruction is performed through a bilinear interpolation based approach. These systems do not tackle the varying PSF challenge.

A few row-column ultrasound systems were proposed in recent literature to improve image quality. In [13, 14], a system with column-row-parallel architecture was proposed. This system exploits the row-column architecture to achieve linear scaling of interconnection, acquisition and programming time by transmitting a full, 2-D plane wave and receiving either in rows or columns (hence the terms row-parallel and column-parallel). It was demonstrated that such a system can achieve high frame-rates as well as proved improved contrast with a 16 × 16 row-column system. However, this approach is highly optimized for C-mode imaging, and as such exhibited noticeably reduced performance for common B-mode imaging purposes, which we will illustrate later in this study. A number of recent works [12, 15, 16] employed a more standard approach to row-column ultrasound imaging that has demonstrated strong performance for B-mode imaging. In [15], a real-time ultrasound imaging system was introduced that incorporates row-column addressing-based capacitive micromachined ultrasound transducers (CMUTs) for improved data acquisition speed. The system proposed by [12, 16] attempts to improve image quality by utilizing transducer-integrated apodization to reduce the ghost effects in the point spread function, a drawback common to row-column systems. All of these systems have focused on improved hardware design for better image quality, and as such do not take into account the underlying physical properties of row-column imaging (e.g., speckle noise characteristics, spatially varying beam profile characteristics, and measurement sparsity) into account for compensating for these characteristics in image reconstruction. To get the best ultrasound reconstruction possible, the imaging system must be built on a framework capable of addressing all three challenges.

In this research, we propose a compensated row-column ultrasound imaging system (henceforth referred to as CRC-UIS) that builds on the CMUT-based RC system proposed by [15], which we will denote as the baseline RC system in this study. The reconstruction framework in CRC-UIS is based off of Fisher-Tippett multilayered conditional random field (MCRF) model. MCRF, which was proposed by [17], is an extension to the Conditional Random Field (CRF) model. While the CRF model offers the flexibility to incorporate any noise model as well as enable the use of spatially varying PSF (and its ghost artifacts), MCRF adds a layers of certainty, taking the issue of sparsity into account. This way all three challenges are addressed. The proposed system builds on the baseline RC system by incorporating proper characterization of the row-column system as well as a MCRF based optimization into the system.

## Method

**Fig 3:**
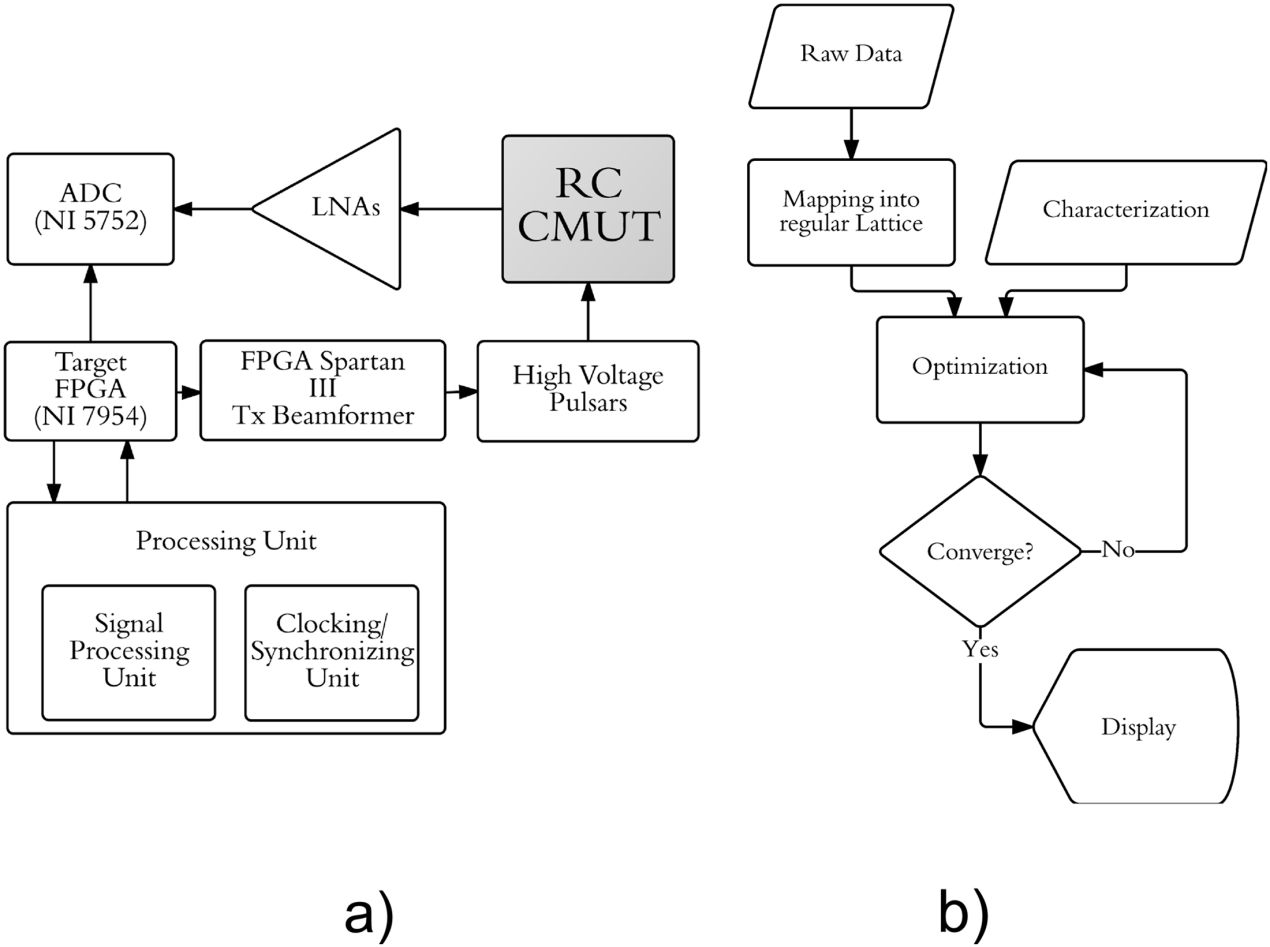
A flow chart representing the proposed system. (a) shows the a top level implementation of the CRC-UIS. (b) shows the signal processing unit in more detail.

Our proposed system has three main stages: data acquisition, characterization, and signal processing. Each stage is presented in detail in this section. A top level implementation of the system can be seen in Fig 3.

### Data Acquisition

The data acquisition unit consists of a customized system built using the PCI eXtensions for Instrumentation (PXI) platform. A row-column addressing capacitive micromachined ultrasonic transducers array (RC-CMUTs) was used, with pre-amplifiers being added to compensate for its small current output signal. A digitizer, an FPGA board, and an embedded controller module are also included. An external FPGA was included for transmit beamforming, as well as a set of high voltage pulsers for stepping up voltage. The system block diagram is shown in Fig 3.

### Characterization

This subsection details the characterization of the intrinsic properties of the data acquisition unit, which will be used as input to the signal processing unit. First we discuss the mathematics of how an image is observed, then we present how noise is modelled, finally we describe how we characterize the PSF at different depths.

#### Image Formation

Eq 1 describes how a true image is observed when the row-column technique is used:

$$g\left(r,\theta ,\phi \right)=M\left(r,\theta ,\phi \right)[f\left(r,\theta ,\phi \right)*h\left(r,\theta ,\phi \right)u_{m}\left(r,\theta ,\phi \right)+u_{a}\left(r,\theta ,\phi \right)]$$ (1)

where *r*, *θ*, and *ϕ* denote the radial distance, the azimuthal angle, and the polar angle respectively. The term *g*(*r*, *θ*, *ϕ*) is the observed image, *M*(*r*, *θ*, *ϕ*) is the sampling function, *f*(*r*, *θ*, *ϕ*) is the tissue reflectivity function, *h*(*r*, *θ*, *ϕ*) is the spatially dependent point spread function (PSF); a function that describes the response of an imaging system to a point source, *u*_*m*_(*r*, *θ*, *ϕ*) is the multiplicative noise, and *u*_*a*_(*r*, *θ*, *ϕ*) is the additive noise. * denotes the convolution operation.

To express Eq (1) in the more common Cartesian form, the spherical coordinates are converted using the equations:

$$\begin{array}{cc} x & =r\sin(\theta )\cos(\phi )\\ y & =r\sin(\theta )\sin(\phi )\\ z & =r\cos(\theta ) \end{array}$$ (2)

where x, y, and z are the Cartesian coordinates.

Using Eqs (2) and (1) can be expressed as:

$$g\left(x,y,z\right)=M\left(x,y,z\right)[f\left(x,y,z\right)*h\left(x,y,z\right)u_{m}\left(x,y,z\right)+u_{a}\left(x,y,z\right)].$$ (3)

**Fig 4:**
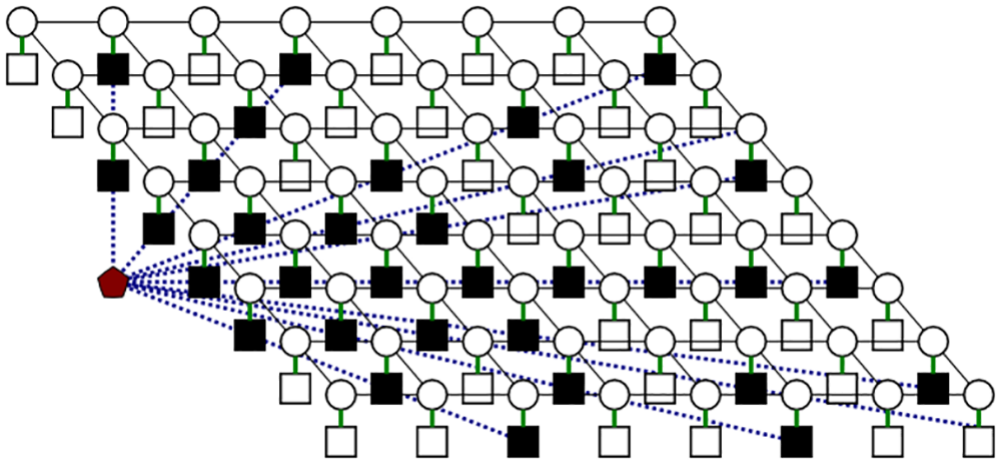
Fan beams originating from the ultrasound transducer. Black squares indicate available readings, white squares indicate absent readings that need to be estimated.

The observed image *g* is a series of fan-beams of ‘readings’, originating from the ultrasound source, in a three dimensional black box. This is illustrated in Fig 4, where the black squares show the available readings.

#### Noise Model

Scans from all coherent imaging modalities present with Speckle noise. This noise is a byproduct of the interfering echoes of a transmitted waveform that emanate from the studied object’s heterogeneities. Noise in ultrasound images is often modeled as:

$$g\left(x,y,z\right)=f\left(x,y,z\right)\xi_{m}\left(x,y,z\right)+\xi_{a}\left(x,y,z\right)$$ (4)

where *g*(*x*, *y*, *z*) is the noisy observation, *f*(*x*, *y*, *z*) is the noise-free image, *ξ*_*m*_(*x*, *y*, *z*) is the multiplicative noise due to coherent interference (often referred to as speckle), and *ξ*_*a*_(*x*, *y*, *z*) is the additive noise (sensor noise, etc.).

This model mirrors the image formation model in Eq (1), with the exception of the sampling function *M*(*x*, *y*, *z*) and the PSF *h*(*x*, *y*, *z*). The noise components from the image formation model will now be considered.

Additive noise is considerably smaller than the multiplicative one. Therefore, the additive noise term can be removed from Eq (3) and *g*(*x*, *y*, *z*) can be expressed as:

$$g\left(x,y,z\right)=M\left(x,y,z\right)f\left(x,y,z\right)h\left(x,y,z\right)u_{m}\left(x,y,z\right).$$ (5)

Taking the log of Eq (5) would turn the multiplication into a simple addition problem:

$$\log\left(g\left(x,y,z\right)\right)=\log\left(M\left(x,y,z\right)\right)+\log\left(f\left(x,y,z\right)\right)+\log\left(h\left(x,y,z\right)\right)+\log\left(u_{m}\left(x,y,z\right)\right).$$ (6)

Following the assumption that the multiplicative noise in Eq (5) follows the Generalized Gamma distribution [18], the noise samples of the logarithmic transformed multiplacitive noise in Eq (6) can be modled with the Fisher–Tippett distribution given by:

$$p\left(I\left(x,y,z\right)\right)=2\exp[\left(2I\left(x,y,z\right)-\ln2\sigma^{2}\right)-\exp\left[2I\left(x,y,z\right)-\ln2\sigma^{2}\right]]$$ (7)

where *p* is the probability density function (PDF), *I*(*x*, *y*, *z*) denotes voxel intensity at point (*x*, *y*, *z*), and *σ* is their standard deviation.

The spatially varying PSF presented in Eq (1), the sparsity due to the sampling function also presented in Eq (1), and the noise model in Eq (7) can all be incorporated into a MCRF based model. The details of their mathematical incorporation will be presented in the signal processing subsection.

#### PSF Characterization

The PSF characterization was done independently from the data acquisition unit, but was incorporated along with the acquired data as input to the signal processing unit. The characterization was achieved using an open source MATLAB toolkit called Field II [19], where the parameters of the imaging system can be defined to find an estimate of the PSF at any point away from the transducer. The model in Field II, shown in Eq (8), is based on Tuphole and Stephanishen’s spatial impulse concept inherited from the linear systems theory [20]. For a particular transducer geometry *S* with field point $\vec{r_{1}}$ and transducer position $\vec{r_{2}}$, the spatially dependant three dimensional point spread function can be expressed as:

$$h\left(\vec{r_{1}},t\right)=\int_{S}\frac{\delta \left(t-\frac{|\vec{r_{1}}-\vec{r_{2}}|}{c}\right)}{2\pi |\vec{r_{1}}-\vec{r_{2}}|}dS$$ (8)

where *δ* is the Dirac delta function, *t* is time, and *c* is the speed of sound at homogeneous medium of density *ρ*_0_.

### Signal Processing Unit

Fig 3 shows how the signal processing unit contributes to the overall system. This unit is the framework that drives the reconstruction of the ultrasound image. It incorporates the intrinsic properties of ultrasound as well as the acquired raw data into a MCRF framework capable of addressing the three challenges the baseline RC system could not. Raw data is mapped into a regular lattice and passed on to the optimization algorithm, once it converges the resulting image is displayed. The mathematical expression that drives this optimization will now be formulated.

#### MCRF Formulation

To estimate the tissue reflectivity function *f*(*x*, *y*, *z*), the inverse problem of Eq (3) needs to be solved. The relationship between observed image and actual signal can be modeled as a conditional probability of true signal given the observation. We can formulate the reconstruction problem as a Maximum a Posteriori (MAP) estimation problem [21–23], where a solution is obtained by maximizing the posterior distribution:

$$F^{*}=\underset{\bar{F}}{argmax}\left\{P(F|G)\right\}$$ (9)

where F*, $\bar{F}$, and *G* are the MAP solution, the possible results set, and the observation respectively.

Conditional random field (CRF) is a powerful discriminative modeling method, first proposed by [24], that can directly model the conditional probability *P*(*F*|*G*) without specifying any prior model *P*(*F*) and relaxing the conditional independence assumption *P*(*G*|*F*) [17]. The CRF model can be expressed as:

$$P\left(F|G\right)=\frac{1}{Z(G)}\exp\left(-\psi (F,G)\right)$$ (10)

where *Z* is the partition function and *ψ*(⋅) is the potential function [17, 24–28]. The potential function *ψ*(⋅) is the combination of any arbitrary unary *ψ*_*u*_(⋅) and pairwise *ψ*_*p*_(⋅) potential functions:

$$\psi \left(F,G\right)=\sum_{i=1}^{n}\psi_{u}\left(f_{i},G\right)+\sum_{c\in C}\psi_{p}\left(f_{c},G\right)$$ (11)

where *C* is a set of a clique structure corresponding to the Markov neighborhood [25].

**Fig 5:**
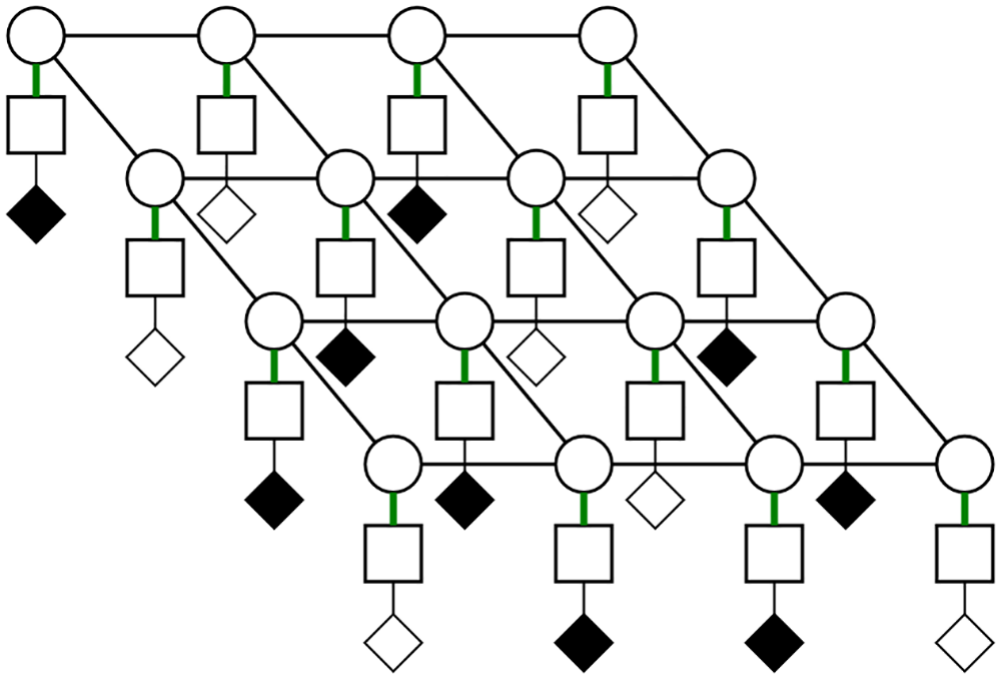
Visualization of the uncertainty layer within the state-observation model. This is a 2-D slice of the full 3-D lattice. The layers (from top to bottom) are state, observation, and uncertainty layer.

Regular CRFs adopt local cliques (or neighborhoods) where random variable interactions are involved in modeling. In this conventional framework, neighbours are considered with the same degree of certainty. To put it more simply: CRFs assume observations are complete; they don’t take data sparsity into account. However, one of the challenges this framework aims to address is to reconstruct a full 3-D volume *F* from a set of sparse measurements *G*. MCRF introduces an extension to the CRF model where a layer that determines the degree of the observation’s uncertainty is incorporated, thereby addressing the issue of incomplete data. With MCRF, every observation is linked with a value that specifies the uncertainty in modeling. With this extension, Eq (10) can be rewritten as:

$$P\left(F|Cr,G\right)=\frac{1}{Z(G)}\exp\left(-\psi (F|Cr,G)\right)$$ (12)

where *Cr* is the model’s uncertainty layer. *Cr* is a zero-one plane where *Cr* = 1 at positions with missing observations and *Cr* = 0 at positions where observations are available. Fig 5 demonstrates this layer in context with states and observations (where missing observations are black in this layer). This layer must be taken into account when the unary and pairwise functions are chosen.

**The unary potential function** plays the role of data-driven procedure, incorporating the information corresponding to the observation into the model. Since we believe that the observation is degraded according to the distribution shown in Eq (7), Fisher–Tippett noise is assumed as the degradation process and is incorporated in to the model as the unary potential function:

$$\psi_{u}\left(f_{i},G,Cr_{i}\right)=\left\{\begin{array}{cc} \Psi (f_{i},G), & \;Cr_{i}=0\\ 0 & \;Cr_{i}=1 \end{array}\right.$$ (13)

where Ψ(*f*_*i*_, *G*) is expressed as:

$$\Psi \left(f_{i},G\right)=\frac{1}{\sigma }\exp\left(-\alpha \frac{\log G-\log H(f_{i})}{\sigma }\right).\exp\left(-\frac{\log G-\log H(f_{i})}{\sigma }\right)$$ (14)

where H denotes the function taking factors related to the imaging system (such as the spatially dependant PSF, sensor noise, etc.) into account, and *α* is the coefficient that determines the contribution of the observed data inside the ‘beams of readings’. The expression for Ψ(*f*_*i*_, *G*) comes from the Generalized Extreme Value theorem, which simplifies to the Fisher–Tippett PDF expressed by Eq (7).

**Fig 6:**
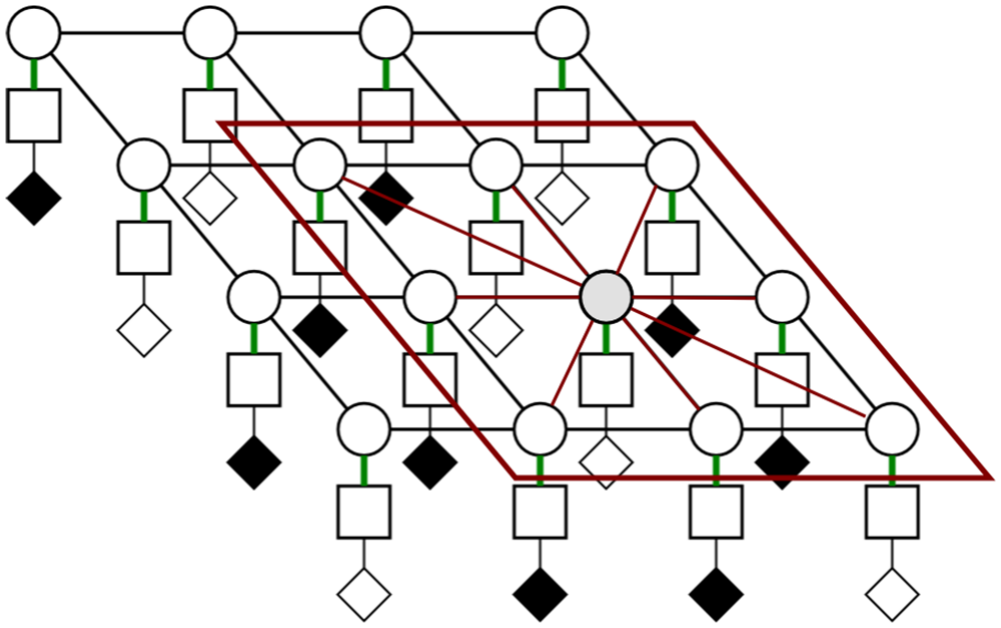
Pairwise relationship illustrated in the state-observation-uncertainty model. The red window shows a clique, and pairwise connectivity of points within that clique is shown in red lines.

**The pairwise potential functions** incorporates the spatial information into the model. These functions are defined based on a subset of random variables which is determined by clique structures. This is demonstrated in Fig 6, where according to a predefined penalty function *w*(⋅), the relations among random variables in a clique *c* can be defined as:

$$\psi_{p}\left(f_{c},G\right)=\exp(-\beta |f_{i}-f_{j}\left|w\left(g_{i},g_{j}\right)\right)$$ (15)

where {*i*, *j*}∈*c*, *β* is the coefficient that determines the contribution of the spatial information, and *w*(*g*_*i*_, *g*_*j*_) is the penalty function. Note that *c* is simple clique, not to be confused with the uncertainty layer *Cr*.

The pairwise term aims to remove small noises, provide consistent labels in neighboring random variables and estimate the areas of the image with no prior data with the help of penalty functions based on the spatial information available. The penalty function attempts to use whatever information that is already available to find the best estimate for the ‘dark’ areas of the image. For the penalty function, two penalty terms are included: spatial proximity penalty term *w*_*sp*_ and First Order Variation (FOV) of intensity values *w*_*fov*_.

*The spatial proximity penalty term* is based on the assumption that the farther a voxel is, the less likely it is to belong to a unique segment of an image. It maintains the homogeneity of surrounding voxels. The spatial proximity between voxels *i* and *j* is quantified by the Euclidean distance *d*_*E*_(*i*, *j*):

$$w_{sp}\left(i,j\right)=\exp\left(\frac{-d_{E}\left(i,j\right)}{2\sigma_{sp}^{2}}\right)$$ (16)

where *σ*_*sp*_ is a control factor used to enforce the strength of spatial closeness.

*The first order variation (FOV) penalty term* is built on the need to preserve the boundaries of the estimated image, it uses the difference in intensities between neighbouring voxels to outline tissue transitions and provide a more clear ultrasound image. The penalty term is expressed as:

$$w_{fov}\left(g_{i},g_{j}\right)=\exp\left(-\frac{\left|\right|g_{i}-g_{j}\left|\right|}{2\sigma_{fov}^{2}}\right)$$ (17)

where *σ*_*fov*_ is a control factor used to enforce the strength of this penalty term.

#### Energy Function Inference

Given the MCRF expression in Eq (12) together with the potential function in Eq (11), the energy function for the MAP model can be formulated as:

$$E\left(F,G,Cr\right)=\sum_{i=1}^{n}\psi_{u}\left(f_{i},G,Cr_{i}\right)+\sum_{c\in C}\psi_{p}\left(f_{c},G\right).$$ (18)

The MAP can now be reformulated as:

$$F^{*}=\underset{\bar{F}}{argmin}\left\{E(F,G,Cr)\right\}.$$ (19)

To solve this MAP problem, a gradient descent algorithm was used. Gradient descent is an iterative optimization algorithm that finds the minimum by taking steps that are proportional to the negative of the gradient at a certain point. The gradient descent for possible solution F* can be expressed as:

$${F^{*}}^{t+1}={F^{*}}^{t}+\frac{\nabla E(F,G,Cr)}{\nabla F}$$ (20)

where $\frac{\nabla E(F,G,Cr)}{\nabla F}$ is the energy gradient with respect to *F* and F*^t^ is the estimated solution at iteration *t*. To find the possible solution F* while taking into account the energy function given in Eq (18) and the potential functions given in Eqs (13) and (15), the gradient descent in Eq (20) can be rewritten as:

$${F^{*}}^{t+1}={F^{*}}^{t}+\alpha \left(\frac{\nabla \psi_{u}\left(F,G,Cr\right)}{\nabla F}\right)+\beta \left(\frac{\nabla \psi_{p}\left(F,G\right)}{\nabla F}\right)$$ (21)

where $\frac{\nabla \psi_{u}\left(F,G,Cr\right)}{\nabla F}$ is the gradient of the unary part of the energy function with respect to *F*, $\frac{\nabla \psi_{p}\left(F,G\right)}{\nabla F}$ is the gradient of the pairwise part of the energy function with respect to *F*, *α* determines the contribution of the unary part of the energy function, and *β* determines the contribution of the pairwise part of the energy function.

## Experimental setup

To evaluate the efficacy of our proposed system, CRC-UIS was tested on simulated ultrasound scans as well as real ultrasound scans. Simulated scans were compared against the baseline RC system, the column-row-parallel system [13, 14], the integrated apodization system [12, 16], and a system with a fully-addressed 2-D array. Real scans were only compared against the baseline RC system. Simulations were performed using Field II, and open source MATLAB toolkit that has been used in ultrasound literature [19]. For both simulated and real testes, raw data from the scans was mapped into a regular 3-D lattice through linear interpolation before passing it to the optimization stage of CRC-UIS.

### Simulation

The generation of phantom data, simulation of ultrasound images, and calculation of PSF at different depths were all achieved using Field II.

To create the phantom data, phantom dimensions are first defined. A general scatterer based on these dimensions was made. Amplitudes with a Gaussian distribution were generated, and then a high scattering region was made. The amplitude inside the region of interest (predefined cyst positions and dimensions) was set to ten times the amplitude outside. The x-y-z positions of these amplitudes were recorded to be loaded later.

To generated the simulated data, the transducer apertures were first defined. Apertures for emission and reception were then generated. The impulse response and excitation of the emit and receive aperture were set. Phantom data is then loaded, where beamforming in a manner identical to real row-column imaging devices is performed by Field II.

To model the PSF at a particular depth, the transducer apertures were first defined. Apertures for emission and reception were then generated. A point phantom is created at the required depth, and a linear sweep is then made to calculate the response. A point scatterer was then generated and the PSF at the required spatial location was found.

### Simulated phantom

**Fig 7:**
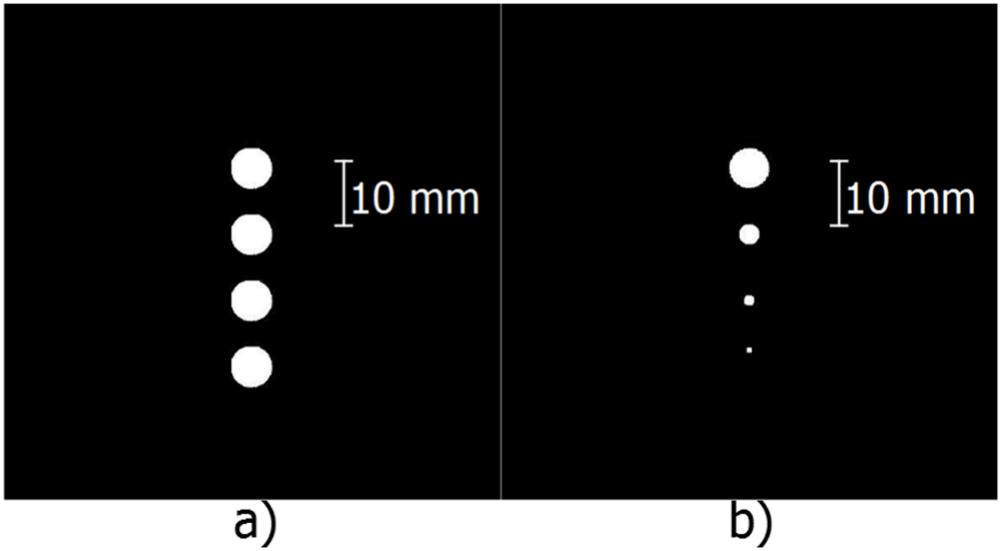
Model of artificial phantoms. (a) shows the first artificial phantom with 4 cysts of equal size, (b) shows the second phantom with 4 cysts of decreasing size.

For the simulated scan, two phantoms (shown in Fig 7) were created. The first phantom consisted of four cysts; 6 mm in diameter and 10 mm apart. The second also consisted of four cysts that are 10 mm apart, but the diameter was gradually reduced.

The first phantom aims to show how one particular shape can vary with depth. The second one aims to show how the reconstruction of a shape differs as the size of that shape changes.

### Real Data

For both the baseline RC system and the proposed CRC-UIS system, the volumetric scanning data was acquired by a customized imaging system built using the PCI eXtensions for Instrumentation (PXI) platform. A row-column addressing capacitive micromachined ultrasonic transducers array (RC-CMUTs) was used. The 32 by 32 two-dimensional array has a center frequency of 5.9 MHz, an aperture size of 4.8 mm by 4.8 mm with a 150 μm pitch. Pre-amplifiers were used since CMUTs have small current output signals. The PXI system includes a 32 channel digitizer (NI-5752, National Instruments), a FPGA board (NI-7954, National Instruments), and an embedded controller module, which includes an Intel Core 2 Quad 2.26 GHz CPU and a Windows 7 operating system. An external FPGA was responsible for transmit beamforming while a set of high-voltage pulsers (LM96551, Texas Instruments) responsible for stepping the voltage to 30 V were used. The CMUTs were biased at -60 V to improve sensitivity and was operated in conventional mode. The system block diagram is shown in Fig 3.

Receive beamforming is done with the classic delay-and-sum method. Hilbert’s transform is used to detect the envelope of the summed signal follow. The depth and angle, both azimuth and elevation, are then processed with the reconstruction framework.

### Real phantom

For the real row-column ultrasound scan, wire target imaging was performed. Four wires, all 644 μm in diameter, were arranged in a way to allow for a scan of their cross sections.

### Algorithm setup

The MCRF reconstruction framework for CRC-UIS was implemented using MATLAB software. The algorithm was first performed several times to identify the optimal values for the control parameters in the MCRF (as defined in section III). Empirical testing led to the choice of *σ*_*sp*_ = 5 for controlling spatial proximity and *σ*_*fov*_ = 0.03 for controlling first order penalty term. The clique size was chosen to be 11 × 11 pixels. Furthermore, for the simulated data, *α* = 0.3 and *β* = 1.0 were chosen as the weighted contributions of unary and pairwise functions respectively. For real data *α* = 0.7 and *β* = 0.3 were the best choices. In this study, the MCRF reconstruction framework for CRC-UIS reconstructs each slice of the 3-D volume independently to form the final 3D image volume; nevertheless, it does account for spatial beam profile variations in 3D volumetric space.

### Metrics for comparison

For the purpose of our implementation, Peak Signal to Noise Ratio (PSNR), Effective Number of Looks (ENL), and Coefficient of Correlation (CoC) were used as metrics to evaluate the performance of our framework on simulated data. Signal to Noise Ratio (SNR) and ENL metrics were used to evaluate the performance of our framework on real data. All metrics were defined according to recent literature [29–35].

PSNR is a metric that provides quality measure in terms of the power of the ideal and reconstructed image. As shown in Eq (22), its is based on Mean Square Error (MSE) defined in Eq (23). PSNR is frequently used in ultrasound noise despeckling literature to measure the performance of speckle removal [29–35]. Higher PSNR indicate better image quality.

$$PSNR=10log_{10}\left(\frac{{\left(MAX(f_{p})\right)}^{2}}{MSE}\right)$$ (22)

where *f*_*p*_ is the ideal image, *MAX*(*f*_*p*_) is the peak signal of *f*_*p*_, and *MSE* is given by:

$$MSE=\frac{1}{MN}\sum_{i=1}^{M}\sum_{j=1}^{N}{\left(f_{p,ij}-f_{r,ij}\right)}^{2}$$ (23)

where *f*_*r*_ is the reconstructed image.

CoC is a metric that gives a measure of edge preservation. For completely uncorrelated images its value is 0, and for identical images its value is 1. Eq 24 shows the mathematical expression for CoC.

$$CoC=\frac{\sum_{i=1}^{M}\sum_{j=1}^{N}\left(\nabla^{2}f_{p,ij}-{\bar{\nabla^{2}f}}_{p}\right)\left(\nabla^{2}f_{r,ij}-{\bar{\nabla^{2}f}}_{r}\right)}{\sqrt{\sum_{i=1}^{M}\sum_{j=1}^{N}{\left(\nabla^{2}f_{p,ij}-{\bar{\nabla^{2}f}}_{p}\right)}^{2}\sum_{i=1}^{M}\sum_{j=1}^{N}{\left(\nabla^{2}f_{r,ij}-{\bar{\nabla^{2}f}}_{r}\right)}^{2}}}$$ (24)

where ▽^2^ is the laplacian operator $\bar{f}$ is the sample mean:

$$\bar{f}=\frac{1}{MN}\sum_{i=1}^{M}\sum_{j=1}^{N}f_{ij}.$$ (25)

ENL provides a measure of the statistical fluctuations (often introduced by speckle) in a particular region of interest; it gives and idea on how smooth a homogeneous region is. Higher ENL values indicate smoother regions. The mathematical expression for ENL is shown in Eq (26), the ENL value is based on voxel mean *μ*_*t*_ and standard deviation *σ*_*t*_ of the region of interest *t*.

$$ENL=\frac{\mu_{t}^{2}}{\sigma_{t}^{2}}.$$ (26)

SNR, like PSNR, is often used as a measure of the performance of speckle removal. Higher SNR values indicate better image quality. Since ground truth is unavailable for real data, an alternative definition of SNR is used (shown in Eq (27)) where the mean *μ*_*r*_ and variance *σ* ^2^ of the pixels in the reconstructed image are the basis of the definition.

$$SNR=\frac{\mu_{r}}{\sigma_{r}^{2}}.$$ (27)

## Results

To evaluate the performance of our proposed MCRF reconstruction framework, the simulated output images from the CRC-UIS system were compared against simulated output images from the baseline RC system, the column-row-parallel system [13, 14], the integrated apodization system [12, 16], and the fully addressed 2-D array. Real images from the CRC-UIS system were compared against real images from the baseline RC system. The comparison was done both quantitatively as well as visually.

### Quantitative Evaluation

To quantify the performance of our reconstruction framework, metrics defined in recent related studies [29–35] were used. For the simulated data, comparisons were made between the output image and the ideal image; the original phantom image. For the real data, the metrics chosen account for the absence of ground truth.

The results of the CRC-UIS reconstruction were compared against the output of other systems in literature with the ideal phantom image as reference. Table 1 summarizes the results for simulated data. Table 2 summarizes the results of the real phantom.

**Table 1:** Quantitative results for the simulated phantoms. For both the first and second phantoms, it is clear that CRC-UIS outperforms other system when it comes to PSNR and ENL, with the exception of the first phantom in the fully addressed 2-D array, where PSNR values were close.

phantom	System	PSNR (dB)	CoC	ENL
1	CRC-UIS	15.9661	**0.0206**	**11.5362**
	Baseline RC [15]	12.0393	0.0076	7.2600
	Integrated apodization [7]	7.9748	0.0095	11.0534
	Fully addressed 2-D array	**16.1739**	0.0138	0.6250
	Column-row-parallel [13]	1.8939	0.0010	2.6238
2	CRC-UIS	**19.6479**	**0.022**	**12.0280**
	Baseline RC [15]	15.0033	0.0076	0.6303
	Integrated apodization [7]	11.9303	0.0095	3.9362
	Fully addressed 2-D array	10.7894	0.003	0.5697
	Column-row-parallel [13]	2.3644	0.0006	3.4690

**Table 2:** Quantitative results for the real phantom. Similar results to the simulated data tests, where CRC-UIS outperformed the baseline RC system when it comes to SNR and ENL.

System	SNR	ENL
CRC-UIS	**14.4046**	**50.3531**
Baseline RC [15]	8.3206	23.0397

Quantitative analysis of the simulated data show that the proposed CRC-UIS is capable of boosting PSNR and improving ENL of the baseline RC system, as well as performing better than (or close to, in case of the PSNR of the second phantom) the fully addressed 2-D array. Oue proposed system also has the highest CoC score, indicating better edge preservation. These results indicate that our approach is better at suppressing noise, preserving edges, and providing smoother regions inside the cysts and outside it. The results of the integrated apodization system point show better performance in CoC and ENL at the cost of PSNR loss when compared to the baseline RC system, indicating better defined edges as well as smother regions inside and outside the cysts. Our proposed system was still able to provide smoother regions without apodization while maintaining a higher PSNR, but did not perform as well with the edge preservation metric. The column-row-parallel, as mentioned in the introduction section, was not optimized for B-mode scans, the poor performance was not surprising.

Quantitative analysis of the real data show that the proposed CRC-UIS is capable of outperforming the baseline RC system in terms of SNR and ENL. CRC-UIS showed a SNR 6 dB higher, demonstrating the noise suppression part of the framework. CRC-UIS also outperformed the baseline RC system when it came to ENL metric, implying smoother regions both inside the wire cross-section and outside it.

### Visual Evaluation

**Fig 8:**
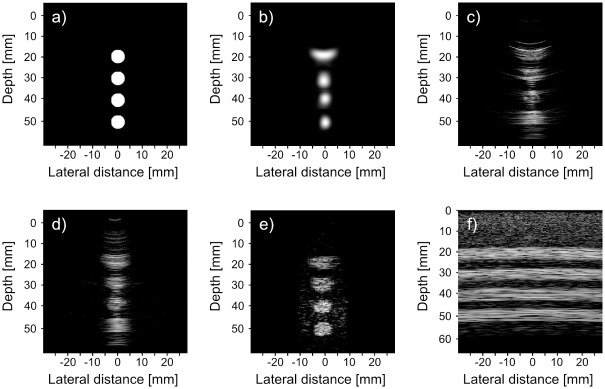
Visual assessment of our proposed CRC-UIS (top center) as opposed to other systems in literature. The first phantom is shown in (a), CRC-UIS reconstruction is shown in (b), Baseline RC system shown in (c) with a dynamic range of 40 dB, integrated apodization system [7] shown in (d) with a dynamic range of 60 dB, fully addressed 2-D array shown in (e) with a dynamic range of 30 dB, and column-row-parallel system [13] shown in (f) with a dynamic range of 30 dB.

**Fig 9:**
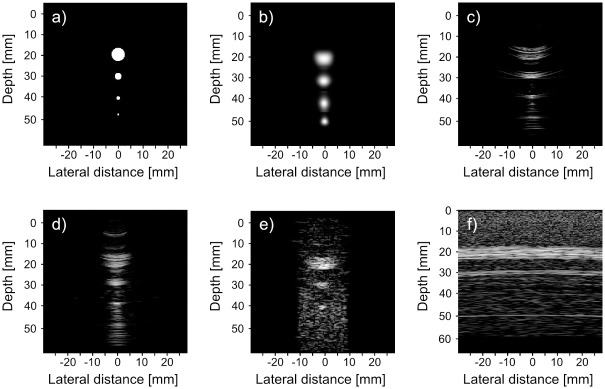
Visual assessment of our proposed CRC-UIS (top center) as opposed to other systems in literature. The second phantom is shown in (a), CRC-UIS reconstruction is shown in (b), Baseline RC system shown in (c) with a dynamic range of 40 dB, integrated apodization system [7] shown in (d) with a dynamic range of 60 dB, fully addressed 2-D array shown in (e) with a dynamic range of 30 dB, and column-row-parallel system [13] shown in (f) with a dynamic range of 30 dB.

Figs 8 and 9 show the reconstruction of the simulated phantom data for CRC-UIS as well as other systems in literature. Visual assessment with both simulated phantoms shows that CRC-UIS presents smoother images with less noise and more preserved edges when compared to other systems. These observations are also supported by the quantitative evaluation.

For the first phantom images in Fig 8, the top cysts in the CRC-UIS reconstruction (the one farthest away from the focus depth and closest to near-field) is deformed and larger than it should be, but the rest of the cysts are equally sized and properly shaped. Only the integrated apodization system and fully addressed 2-D array have four equally sized and properly shaped cysts. However, both images are extremely noisy and the cysts have no clear edge. The baseline RC system has a few visible artifacts and the top and bottom cysts are bigger than they should be. The column-row parallel gives no clear information on the cysts, which is to be expected with vertically placed phantoms as there is no transmit focus.

For the second phantom images in Fig 9, the CRC-UIS reconstruction maintains the proper shape of the cysts and shows the decreasing trend of cysts diameter, although the last the two smaller cysts are larger than the actual size. The fourth cysts cannot be seen in the other systems due to noise, although the fully addressed 2-D array was the closest at maintaining the actual size of the first three cysts.

**Fig 10:**
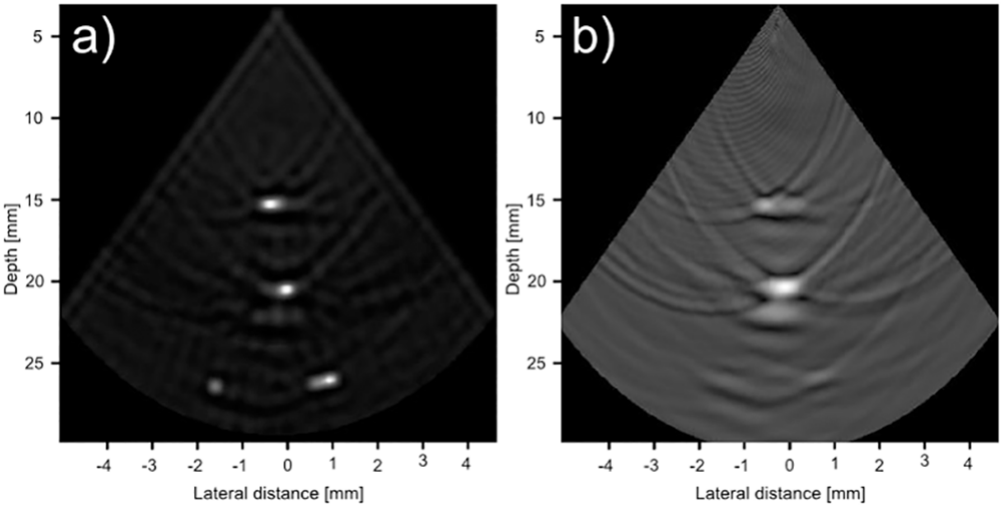
Visual assessment of our proposed CRC-UIS (left side) as opposed to the baseline RC system (right side). CRC-UIS reconstruction shows better noise reduction while maintaining the shape of the phantom. 40 dB is the dynamic range.

**Fig 11:**
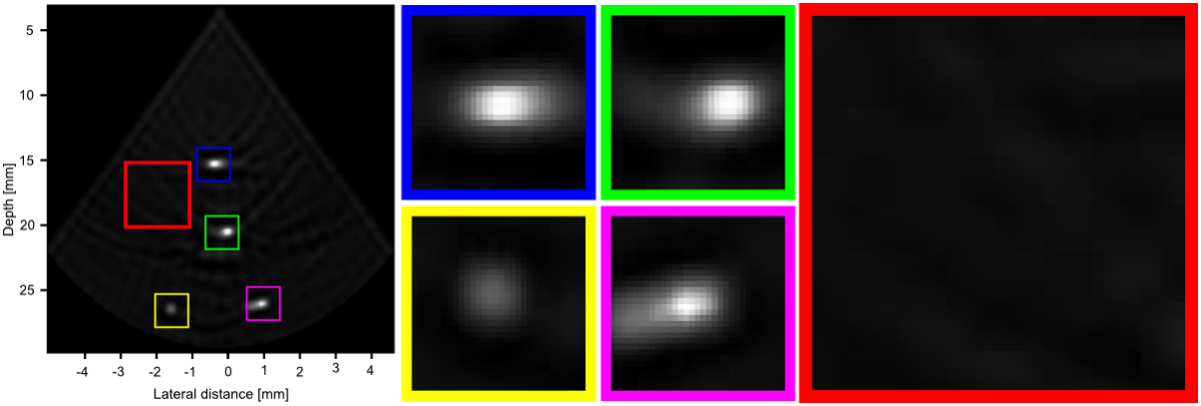
A closer look at the CRC-UIS reconstruction. The four wire targets (shown in blue, green, yellow and pink) have a more consistent shape and size. They are also more clearly visible. A region in the background (shown in red) shows a better suppression of noise when compared to the one in the baseline RC system reconstruction.

**Fig 12:**
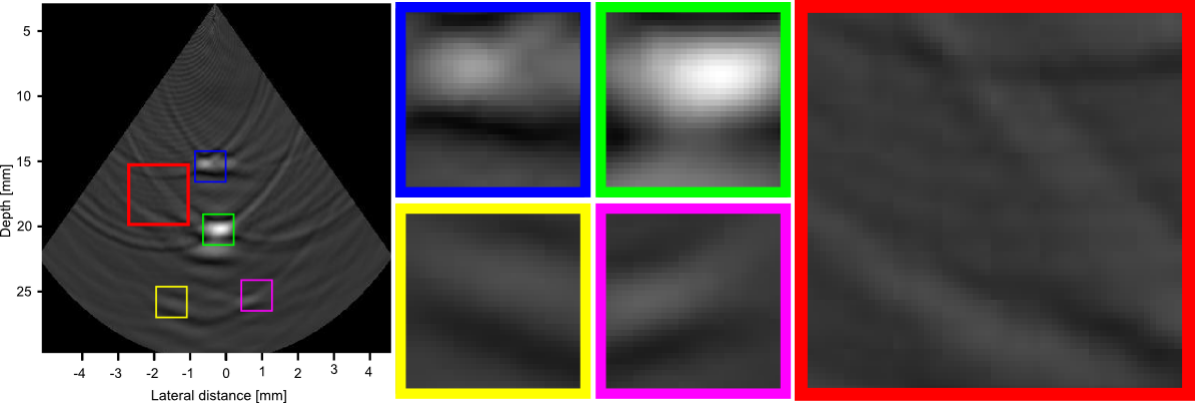
A closer look at the baseline RC system reconstruction. Only two of the four wire targets (blue and green) are clearly visible, and they do not have a consistent shape or size. A region in the background (shown in red) shows unsuppressed ringing noise.

Fig 10 shows the reconstruction of the real phantom data for both CRC-UIS and the baseline RC system. Visual assessment of the real ultrasound image data shows the CRC-UIS was better at suppressing noise, particularly the ringing artifacts noticeable in the baseline RC system’s image output. CRC-UIS was also able to recover all four wires, and maintained a more accurate and consistent shape for all four wires. A more thorough assessment can be see in Fig 11 for the CRC-UIS reconstruction and Fig 12 for the baseline RC system. These observations are supported by the quantitative evaluation.

## Conclusion

### Summary

In this research, we proposed CRC-UIS: a compensated row-column ultrasound imaging system that uses Fisher-Tippett multilayered conditional random field model. This system builds on a baseline RC imaging system and utilizes the intrinsic properties of the data acquisition unit as well as ultrasound into a MCRF based optimization model. This model accounts for missing data, is spatially dependant to incorporate the changing PSF caused by the varying beam profile, and accounts for speckle noise inherent to ultrasound. Through visual analysis of both simulated and real ultrasound images, our proposed system demonstrated the ability suppress speckle and preserve edges when compared to the baseline RC system. This was supported by quantitative analysis, with our proposed system outperforming other systems in literature when compared under the PSNR, CoC and ENL metrics for simulated data tests, as well as SNR and ENL metrics for real data tests. Our proposed system readily supports applications with real-time image acquisition but no real-time feedback.

### Future Work

There are several directions to pursue in the future. First, for the current study, the MCRF reconstruction framework for CRC-UIS reconstructs each slice of the 3-D volume independently to form the final 3D image volume. Therefore, in the future we aim to extend the framework to adopt a full 3-D optimization in an efficient and effective way, which could have the potential for further improving image quality. Second, we aim to explore more comprehensive comparisons with other row-column imaging systems proposed in literature, which would necessitate the construction of these systems. Third, we will explore other random-field approaches for ultrasound image reconstruction.

## References

[1] Koch A, Gruber S, Scharrer T, Fendt KT, Lerch R, Ermert H. 2D transmission imaging with a crossed-array configuration for defect detection. 2012 IEEE International Ultrasonics Symposium. 2012 Oct;p. 36–39.

[2] Osman A, Hassler U, Kaftandjian V, Hornegger J. An automated data processing method dedicated to 3D ultrasonic non destructive testing of composite pieces. IOP Conference Series: Materials Science and Engineering. 2012;42 10.1088/1757-899X/42/1/012005

[3] Smith RA, Nelson LJ. 2D transmission imaging with a crossed-array configuration for defect detection. Insight—Journal of The British Institute of NDT. 2009;51:82 – 87.

[4] Szabo TL. Diagnostic ultrasound imaging: inside out 200 Wheeler Road, 6th Floor, Burlington, MA 01803, USA: Elsevier Academic Press; 2004.

[5] Demore CEM, Joyce A, Wall K, Lockwood GR. Real-time volume imaging using a crossed electrode array. IEEE Transactions on Ultrasonics, Ferroelectrics, and Frequency Control. 2009 6;56(6):1252–1261. 10.1109/TUFFC.2009.1167 19574133

[6] Fritsch C, Parrilla M, Ibanez A, Giacchetta RC, Martinez O. The progressive focusing correction technique for ultrasound beamforming. IEEE Transactions on Ultrasonics, Ferroelectrics, and Frequency Control. 2006 10;53(10):1820–1831. 10.1109/TUFFC.2006.114 17036790

[7] Rasmussen MF, Jensen JA. 3-D ultrasound imaging performance of a row-column addressed 2-D array transducer: A measurement study. IEEE International Ultrasonics Symposium. 2013 July;p. 1460–1463.

[8] Logan AS. The Design, Fabrication and Characterization of Capacitive Micromachined Ultrasonic Transducers for Imaging Applications. University of Waterloo 200 University Avenue West, Waterloo, ON N2L 3G1; 2010.

[9] Logan A, Wong LL, Yeow JTW. 2-D CMUT wafer bonded imaging arrays with a row-column addressing scheme. IEEE International Ultrasonics Symposium. 2009 Sept;p. 984–987.

[10] Logan A, Wong LLP, Chen A, Yeow JTW. A 32 × 32 element row-column addressed capacitive micromachined ultrasonic transducer. IEEE Transactions on Ultrasonics, Ferroelectrics, and Frequency Control. 2011 6;58(6):1266–1271. 21693409 10.1109/TUFFC.2011.1937

[11] Morton CE, Lockwood GR. Theoretical assessment of a crossed electrode 2-D array for 3-D imaging. IEEE Symposium on Ultrasonics. 2003 Oct;1:968–971.

[12] Rasmussen MF, Christiansen TL, Thomsen EV, Jensen JA. 3-D imaging using row-column-addressed arrays with integrated apodization—part i: apodization design and line element beamforming. Ultrasonics, Ferroelectrics, and Frequency Control, IEEE Transactions on. 2015 5;62(5):947–958. 10.1109/TUFFC.2014.006531 25974918

[13] Chen K, Lee BC, Thomenius K, Khuri-Yakub BT, Lee HS, Sodini CG. A column-row-parallel ultrasound imaging architecture for 3d plane-wave imaging and Tx 2nd-order harmonic distortion (HD2) reduction. In: Ultrasonics Symposium (IUS), 2014 IEEE International; 2014. p. 317–320. 10.1109/TUFFC.2018.2811393 29994734

[14] Chen K, Lee HS, Sodini CG. A Column-Row-Parallel ASIC architecture for 3D wearable / portable medical ultrasonic imaging. In: VLSI Circuits Digest of Technical Papers, 2014 Symposium on; 2014. p. 1–2.

[15] Chen A, Wong LL, Logan A, Yeow JTW. A CMUT-based real-time volumetric ultrasound imaging system with row-column addressing. IEEE International Ultrasonics Symposium. 2011 Oct;p. 1755–1758.

[16] Christiansen TL, Rasmussen MF, Bagge JP, Nordahl Moesner L, Jensen JA, Thomsen EV. 3-D imaging using row-column-addressed arrays with integrated apodization—part ii: transducer fabrication and experimental results. Ultrasonics, Ferroelectrics, and Frequency Control, IEEE Transactions on. 2015 5;62(5):959–971. 10.1109/TUFFC.2014.006819 25974919

[17] Kazemzadeh F, Shafiee MJ, Wong A, Clausi DA. Reconstruction of compressive multispectral sensing data using a multilayered conditional random field approach. SPIE Proceedings. 2014;9217.

[18] Michailovich O, Tannenbaum A. Despeckling of medical ultrasound images. IEEE Transactions on Ultrasonics, Ferroelectrics, and Frequency Control. 2006 1;53(1):64–78. 10.1109/TUFFC.2006.1588392 16471433 PMC3639001

[19] Jensen JA. Field: A Program for Simulating Ultrasound Systems. 10th Nordic-Baltic Conference on Biomedical Imaging Published in Medical & Biological Engineering & Computing. 1996;34:351–353.

[20] Jensen JA. Linear descriptions of ultrasound imaging systems DK-2800 Lyngby,Denmark: Technical University of Denemark; 1999.

[21] Black A, Kohli P, Rother C. Markov random fields for vision and image processing. The MIT Press; 2011.

[22] Dolui S. Variable splitting as a key to efficient image reconstruction. University of Waterloo 200 University Avenue West, Waterloo, ON N2L 3G1; 2012.

[23] Sanches J, Bioucas-Dias JM, Marques JS. Minimum total variation in 3D ultrasound reconstruction. IEEE International Conference on Image Processing. 2005 Sept;3:597–600.

[24] Lafferty JD, McCallum A, Pereira FCN. Conditional Random Fields: Probabilistic Models for Segmenting and Labeling Sequence Data. Proceedings of the Eighteenth International Conference on Machine Learning. 2001;p. 282–289.

[25] Shafiee MJ, Wong A, Siva P, Fieguth P. Efficient Bayesian inference using fully connected conditional random fields with stochastic cliques. In: IEEE Conference on Image Processing; Accepted..

[26] Boroomand A, Wong A, Li E, Cho DS, Ni B, Bizheva K. Multi-penalty conditional random field approach to super-resolved reconstruction of optical coherence tomography images. Biomed Optics Express. 2013;4(10). 10.1364/BOE.4.002032 PMC3799664 24156062

[27] Tanaka K, Kataoka S, Yasuda M. Statistical performance analysis by loopy belief propagation in Bayesian image modeling. Journal of Physics: Conference Series. 2010;233(1).

[28] Yao F, Qian Y, Hu Z, Li J. A novel hyperspectral remote sensing images classification using Gaussian Processes with conditional random fields. International Conference on Intelligent Systems and Knowledge Engineering. 2010;p. 197–202.

[29] Achim A, Bezerianos A, Tsakalides P. Novel Bayesian multiscale method for speckle removal in medical ultrasound images. IEEE Transactions on Medical Imaging. 2001 8;20(8):772–783. 10.1109/42.938245 11513028

[30] Shruthi G, Usha BS, Sandya S. Article: A Novel Approach for Speckle Reduction and Enhancement of Ultrasound Images. International Journal of Computer Applications. 2012 5;45(20):14–20.

[31] Wu S, Zhu Q, Xie Y. Evaluation of various speckle reduction filters on medical ultrasound images. Engineering in Medicine and Biology Society. 2013 7;p. 1148–1151. 10.1109/EMBC.2013.6609709 24109896

[32] Sivakumar R, Gayathri MK, Nedumaran D. Speckle filtering of ultrasound B-Scan Images—a comparative study between spatial and diffusion filters. IEEE Conference on Open Systems. 2010 Dec;p. 80–85.

[33] Nageswari CS, Prabha KH. Despeckle process in ultrasound fetal image using hybrid spatial filters. International Conference on Green Computing, Communication and Conservation of Energy. 2013 Dec;p. 174–179.

[34] Srivastava R, Gupta J, Parthasarthy H. Comparison of PDE based and other techniques for speckle reduction from digitally reconstructed holographic images. Optics and Lasers in Engineering. 2010;48(5):626–635. 10.1016/j.optlaseng.2009.09.012

[35] Michailovich O, Tannenbaum A. Blind Deconvolution of Medical Ultrasound Images: A Parametric Inverse Filtering Approach. IEEE Transactions on Image Processing. 2007 12;16(12):3005–3019. 10.1109/TIP.2007.910179 18092599 PMC3643020

